# CRISPR-mediated technology for seed oil improvement in rapeseed: Challenges and future perspectives

**DOI:** 10.3389/fpls.2023.1086847

**Published:** 2023-03-21

**Authors:** Essa Ali, Kewei Zhang

**Affiliations:** Zhejiang Provincial Key Laboratory of Biotechnology on Specialty Economic Plants, College of Life Sciences, Zhejiang Normal University, Jinhua, Zhejiang, China

**Keywords:** CRISPR, *Brassica napus*, seed oil improvement, SgRNA, off-target effects

## Abstract

Rapeseed not only provide considerable amount of edible oil with high nutritional properties but can also be used as a raw material for biofuel production in many industries. It is therefore in high demand to bring genetic changes in order to fulfill the need of human and of industries. Though traditional breeding techniques such as hybridization and mutagenesis remained the top methods for long time to create improved varieties in oilseed rape. Clustered regularly interspaced short palindromic repeats (CRISPR) is becoming one of the most valuable gene editing technologies that allow precise genome engineering, and open new ways for research in plant functional genomics. Though CRISPR has been used in many other crops for genetic improvement it is expected to be an effective tool for genome editing and molecular design in oilseed rape for seed oil improvement. This mini review will discuss and summarize the past and ongoing research and development in rapeseed in terms of seed oil improvement and fatty acid composition using CRISPR technology. In addition, the factors that hinder the efficiency of this tool and how to eliminate those factors will be briefly summarized. The improvement of CRISPR technology for getting better results in oilseed rape will also be considered here. This minireview will open new windows for researchers in *Brassica napus* oil improvement research and genetic improvement using CRISPR technology.

## Graphical Abstract

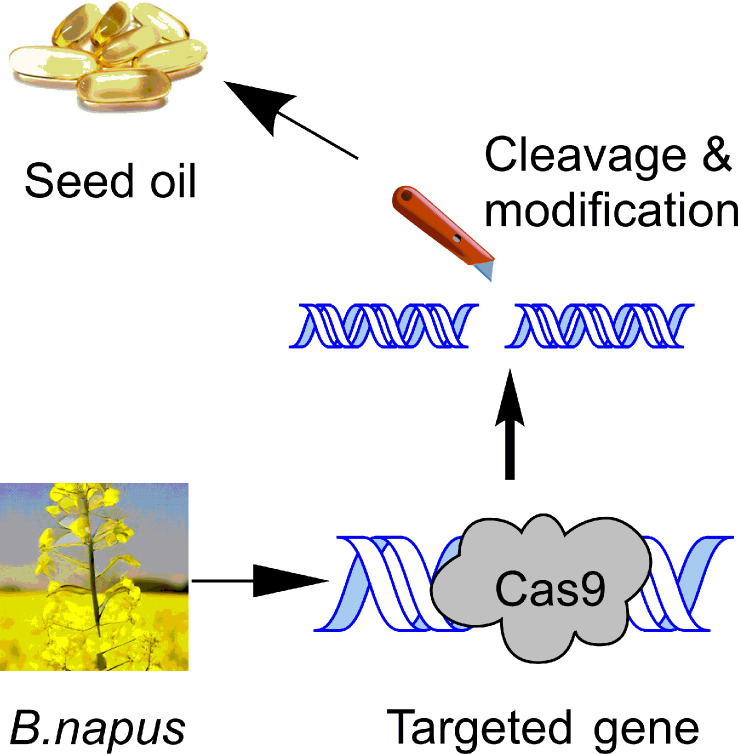

## Introduction

CRISPR/CRISPR-associated (Cas) systems, which were first discovered to give bacteria and archaea adaptive protection against viral and plasmid infection (Barrangou et al., 2007), have been widely used in recent years to modify the genomic DNA in many organisms. Since their inception, the CRISPR-mediated genome editing systems have promised to significantly improve crop genetics with exceptional features (Zhu et al., 2020). CRISPR-based genome editing technique has unmatched advantages over other genome editing systems such ZFN- zinc finger nuclease system and TALEN- transcription activator-like effector nuclease system (Zhang et al., 2017). The quick genetic studies of intricate regulatory circuits by allowing several sgRNAs encoded in a single CRISPR array to delete different target sites is one of those advantages. In addition to deleting genes, it may also be used to insert specific DNA fragments into target sites and specifically change the transcriptional activity of genes (Li et al., 2013; Lowder et al., 2015). Since its initial application in 2013 for plant gene editing, CRISPR/Cas9 technology has been used extensively in food crop species, including rice, wheat, tomato, etc.; industrial crops, including *cichorium intybus*, coffee, dandelion, etc.; ornamental crop species, including lily, lotus, rose, etc.; oil crop species, including rapeseed, soybean, sunflower, etc.; fiber crop (cotton); and feed crop (alfalfa) (Ricroch et al., 2017; Liu et al., 2021). These findings demonstrate the critical role CRISPR/Cas9 is playing in crop improvement and plant functional genomics research.

*Brassica napus*, also known as canola in North America and oilseed rape (OSR) in Europe, the third largest vegetable oil source in the world is grown primarily for its oil-rich seed. The meal produced as a byproduct is utilized as animal feed, primarily in poultry and dairy industries (Wallenhammar et al., 2022). China alone contributes about 20% of the total world production. It is also cultivated in other countries like India, Canada, and the European Union (Hu et al., 2017). For centuries rapeseed farmers have relied on traditional breeding methods in an effort to develop better varieties. Contrarily, *B. napus* is an allotetraploid species with a number of repetitive sequences. Gene redundancy makes it very inefficient to alter traits using traditional genetic methods like hybridization and random mutation. However, gene editing technologies may assist to speed up the process. A CRISPR-Cas9 strategy was successfully used in *B. oleracea* for genome editing, indicating that a transfer to oilseed rape might also be possible (Lawrenson et al., 2015). To prevent seed loss by boosting shatter resistance, the CRISPR/Cas9 technology was initially used in rapeseed in 2017 to target two *BnALC* homologous genes for site-directed mutagenesis (Braatz et al., 2017). The last five years have seen significant agronomical trait advancements in rapeseed using CRISPR/Cas9-mediated genome editing. As of 2022, numerous studies on the effective use of CRISPR/Cas9 technology in *Brassica napus* breeding have been published (Zhang et al., 2019; Yan et al., 2021; Liu Y. et al., 2022; Tan et al., 2022). Indicating the application of this genome editing technology in rapeseed is becoming even more mature and has been extensively used in the creation of germplasm resources and genetic improvement of oilseed rape. CRISPR/Cas9 technology has become an important tool to analyze gene function and molecular mechanisms in rapeseed. We aim to provide pioneer research and will indicate the path for further application of this technology in seed oil improvement in this mini-review.

## Application of CRISPR/Cas9 technology in *Brassica napus*

**Figure 1:**
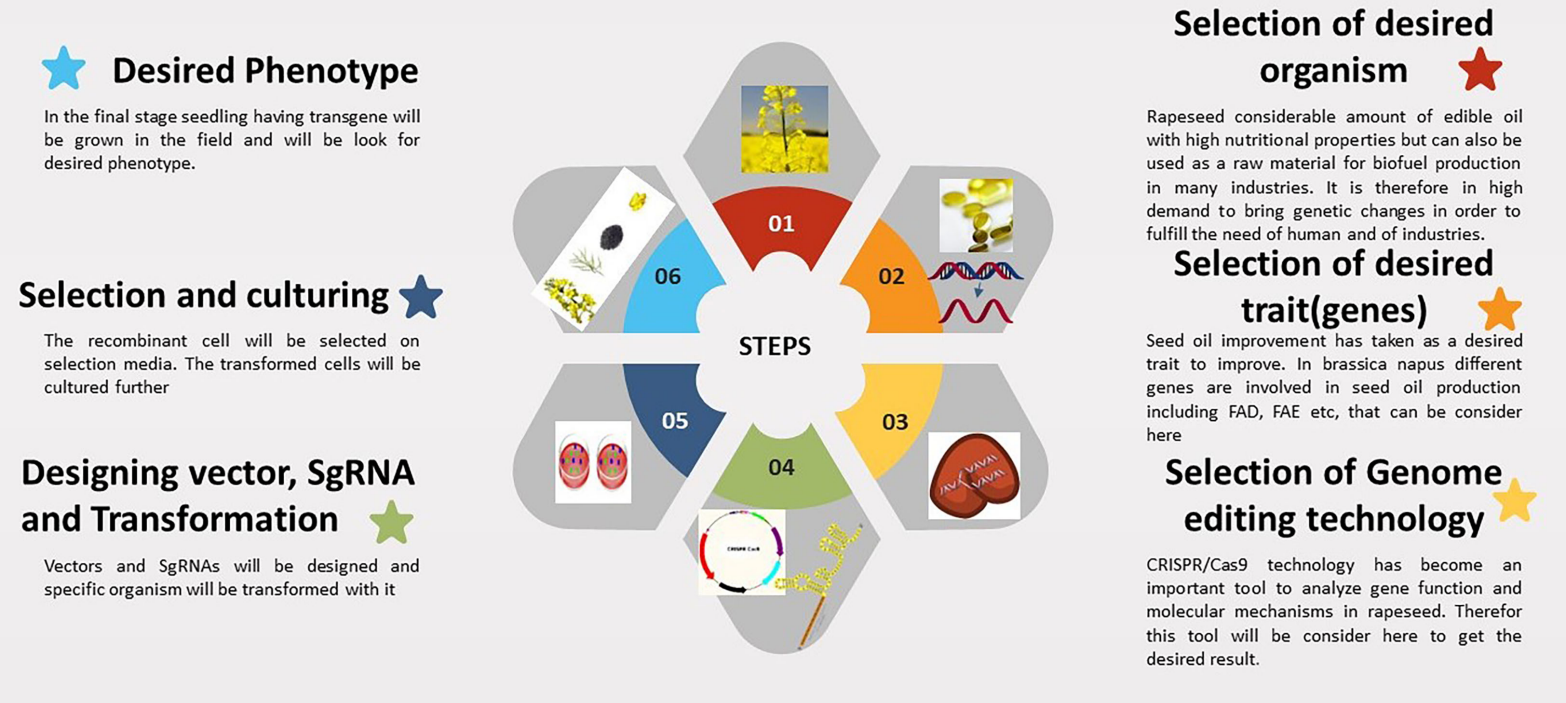
The steps involved in CRISPR technology for seed oil improvement in *Brassica napus*.

The mechanism of CRISPR/Cas9 technology is briefly highlighted in **Figure 1**. *Brassica napus* is an allotetraploid species, and the majority of its genes have redundant functions and multiple copies (Chalhoub et al., 2014). It is exceedingly inefficient to alter features by random mutations because of gene redundancy. To enhance one feature, it is frequently required to modify several genes. The Cas9 protein can be directed to specific regions by a variety of single-guide RNAs (sgRNAs), which presents an opportunity to carry out multiple gene editing by expressing Cas9 along with the various sgRNAs (Cong et al., 2013; Lawrenson et al., 2015). Because numerous mutations can be caused simultaneously in polyploid rapeseed, CRISPR/Cas9 technology provides clear advantages. There are currently a growing number of research using CRISPR/Cas9 to knock out multiple copies of genes in *B. napus*, demonstrating how promising this technology is for understanding of polyploidy in this organism. Whether using a single or multiplex genome editing strategy, the CRISPR/Cas9 system is a useful tool and is frequently employed for crop improvement (Lohani et al., 2020). Multiple gene mutants are typically needed when analyzing the functions of homologous genes and members of gene families with high sequence similarity. Due to their ability to effectively produce mutants with numerous gene mutations, distinct sgRNAs connected in tandem exhibit a significant advantage in multiple gene editing (Xiong et al., 2019). It is also of prime concern that how specific CRISPR/Cas9 technology is when used for targeted gene editing in plants. Typically, any off-target locations should be examined to guarantee that mutation is as specific as desired.

## Main genes edited by Cas technology for seed oil improvement in *B. napus*

**Figure 2:**
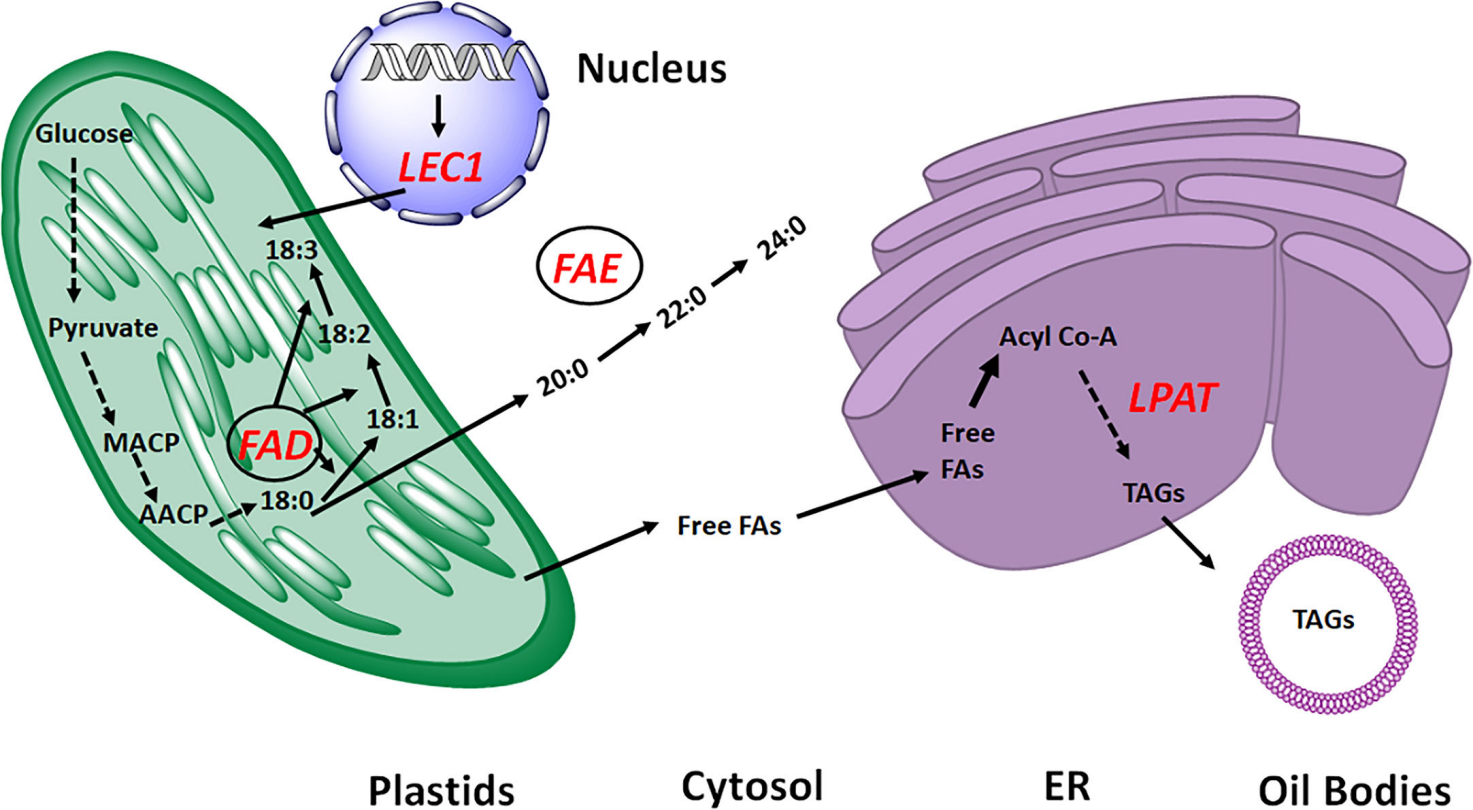
Overview of Fatty acid biosynthesis pathway. The genes targeted for seed oil improvement in *Brassica napus* by CRISPR technology are highlighted as red. FFAs, free fatty acids; MACP, Malonyle ACP; AACP, Acyle ACP; *LPAT*, Lysophosphatidic acid acyltransferase; *FAE*, Fatty acid elongase; *LEC1*, Leafy cotyledon; *FAD*, Fatty acid desaturase; ER, Endoplasmic Reticulum.

Rapeseed breeding has historically focused on improving the fatty acid (FA) composition and increased oil content. Since the introduction of CRISPR/Cas9 in 2017 in rapeseed, the research on genome editing in this species has expanded (Braatz et al., 2017). After that, a significant number of rapeseed genes were modified, creating a useful germplasm resource for studying the fundamental biology of rapeseed and cultivating novel varieties (**Table 1**; **Figure 2**).

**Table 1:** The genes targeted for seed oil improvement in *Brassica napus* L. by CRISPR technology.

Targeted gene	Effects	Reference
*BnFAD2*	Increased oleic acid content	(Okuzaki et al., 2018; Huang et al., 2020)
*BnTT8*	Yellow-seeded traits	(Zhai et al., 2020)
*BnTT2*	Yellow-seeded traits	(Xie et al., 2020)
*BnGTR2*	high oil content	(Tan et al., 2022)
*BnLPAT2*	Enlarged oil bodies and low oil content	(Zhang et al., 2019)
*BnLPAT5*	Enlarged oil bodies and low oil content	(Zhang et al., 2019)
*BnFAE*	Low Erucic acid	(Liu Y. et al., 2022)
*BnLEC1*	Reduced seed oil content and C18:1, increase of C18:2	(Yan et al., 2021)

## Lysophosphatidic acid acyltransferase

The Kennedy pathway’s key enzyme, lysophosphatidic acid acyltransferase (*LPAT*), catalyzes the conversion of FAs into triacylglycerol (TAG), which encourages more oil production (Chapman and Ohlrogge, 2012). Using CRISPR/Cas9, all seven *BnLPAT2* homologous copies and four *BnLPAT5* homologous copies were eliminated to examine their respective functions in the rapeseed oil biosynthesis. The oil content was reduced to diverse degrees in the knockout mutants of the various copies of *BnLPAT2* and *BnLPAT5*, with a 32% decrease in *Bnlpat2* lines, a 29% decrease in *Bnlpat5* lines, and a 39% decrease in *Bnlpat2/Bnlpat5* double mutant lines. This study confirmed that all *BnLPAT* copies could change the oil content, and that oil bodies with knockouts of all copies were larger and contained less oil (Zhang et al., 2019).

## Transparent testa

For instance, by knocking out the *Brassica napus* Transparent Testa-8 (*BnTT8*) gene by using CRISPR/Cas9 technology, yellow-seeded mutants with increased seed oil and protein content as well as altered fatty acid (FA) composition were created. Transparent testa 8 (*TT8*), according to mounting evidence, controls the accumulation of flavonoids in a variety of crops including *Medicago truncatula, Lotus species (* Li et al., 2016; Escaray et al., 2017). It has been determined that the *B. napus* transparent testa 2 (*BnTT2*) allelic variation in the C genome *(BnaC)* plays a role in rapeseed FA production and seed color development (Zhou et al., 2016). Yellow-seeded mutants were produced using the CRISPR/Cas9 technique by knocking out *BnTT8* and *BnTT2* homologs (Xie et al., 2020; Zhai et al., 2020), which displayed enhanced seed oil and changed FA composition. Only two yellow-seeded mutants, *BnTT8* and *BnTT2*, have been produced so far. It is highly encouraging to produce additional yellow-seeded mutants by selectively knocking out essential flavonoid pathway genes.

## Fatty acid desaturase 2

One of the main priorities for genetic manipulation in oilseed rape, is fatty acid content, which influences the nutritional and manufacturing quality of vegetable oil. In oilseed rape, oleic (18:1), linoleic (18:2), and linolenic acid (18:3), synthesis is influenced by the fatty acid desaturase 2 gene (*FAD2*). The allotetraploid rapeseed contains four copies of *BnFAD2*. All the copies of *BnFAD2* were altered using CRISPR/Cas9-based genome editing technology to produce novel allelic variants in oleic acid and other fatty acid levels. Two targeting sites produced a large number of mutants, and the phenotypic diversity of the mutants was extensively assessed. Compared to the wild type’s 66%, the oleic acid content of the mutant seeds grew dramatically, reaching a maximum of 80%, while the levels of linoleic and linolenic acids declined. Compared to *BnFAD2* mutations, *BnFAD2.A5* mutations led to more significant changes in the fatty acid profile. Additionally, mixing various *BnFAD2* mutant alleles can even drastically widen the variety. It was discovered that the impact of various mutation types at *BnFAD2* alleles on oleic acid levels varied, indicating a potential for fatty acid level manipulation by precise mutation at specific region of a gene (Huang et al., 2020). A recent study on *BnFAD2* showed that the CRISPR/Cas9-mediated genome editing of the *BnFAD2*’s double loci (A5 and C5) can precisely edit the targeted genes, increasing the seed’s oleic acid content to a much greater extent than can a single locus-editing technique (Liu H. et al., 2022). Recent findings also reveal that induced mutations in the *BnFAD2* genes by CRISPR/Cas9-mediated editing has produce high oleic acid germplasm from the CY2 cultivar (Shi et al., 2022).

## Leafy cotyledon1

Leafy cotyledon1 (*LEC1*) controls more than 50% of the genes in Arabidopsis involved in plastid FA production (Mu et al., 2008), demonstrating its importance as a transcriptional activator for controlling seed oil content. In *Brassica napus*, though some signal has also seen in the cytoplasm, *BnLEC1* is primarily confined to the nucleus. The two *BnLEC1* homologs have comparable expression patterns, with expression rising during seed development and peaking at 23 DAP. Additionally, the high-oil content line had higher *BnLEC1* gene expression than the low-oil content. The decreasing and increasing pattern of seed oil content were caused by overexpressing and knocking out *BnLEC1*, respectively, demonstrating that *LEC1* homologs have conserved functions in positively regulating seed oil content (Yan et al., 2021).

## Fatty acid elongase 1

Vegetable oils’ processing and edible qualities are influenced by the fatty acid composition, and erucic acid is the fatty acid that may be harmful to the human health. Low erucic acid has therefore always been a breeding characteristic of *B. napus*. In the synthesis of erucic acid, fatty acid elongase 1 (*FAE1*) is essential. Three *B. napus* germplasms with high erucic acid (>30%) and high oil (>50%), the CRISPR/Cas9 technology was employed to produce targeted mutations on two homologous copies of *BnFAE1*. The double mutation of *BnA08.FAE1* and *BnC03.FAE1* (a08c03), resulted in nearly zero erucic acid in all *BnFAE1*-edited germplasms, and the level of oleic acid was increased. This demonstrate that the erucic acid content was significantly reduced by more than ten percent (10%) in the mutant of *BnC03.FAE1 (c03)*. Furthermore, elimination of *BnA08.FAE1* or/and *BnC03.FAE1* had no appreciable impact on other agronomic parameters but marginally reduced seed oil content. These results showed that crisper based technology can be successfully use in producing low erucic acid germplasms of *B. napus*, which offers a workable method for upcoming low erucic acid breeding (Liu Y. et al., 2022). In another study, CRISPR/Cas9 mediated genome editing was used to generate novel knockout plants of *BnFAE1* genes in order to improve the nutritional quality of rapeseed cultivar CY2. As a result a significant increase in oleic acid (70–80%) content was shown in new lines (Shi et al., 2022).

## Challenges and their possible solution

Off-target effects can result in unwanted outcomes even though they are not as serious for CRISPR/Cas applications in plants, still removing off-target effects is always necessary for genome editing. Off-target effects can be greatly reduced by direct delivery of the protein or RNA of Cas enzymes and sgRNAs, such as by RNP transformation. This is due to the fact that these functional molecules are not integrated into plant genomes; as a result, they can swiftly decay after performing their roles and only have a short life in plant cells, which reduces their ability to target additional areas (Zhang et al., 2021). In addition, utilizing the whole twenty three nucleotide (protospacer plus PAM) sequence as a query in the Brassica genome using BLAST searches in conjunction with other CRISPR/Cas9 online resources can enable the selection of guide RNA with no or a low number of projected off-targets (Lawrenson et al., 2019). According to recent research, precision sgRNA design can be used to combat off-target mutations (Jacinto et al., 2020). To prevent unexpected results, it is crucial to design sgRNAs with a minimum amount of off-target activity. Currently, a number of free online prediction tools, like CRISPOR (http://crispor.org) and CCTop (http://crispr.cos.uni-heidelberg.de) (Stemmer et al., 2017; Concordet and Haeussler, 2018), have been utilized to assist researchers in designing sgRNA in an effort to minimize off-target effects. Additionally, a number of machine learning-based techniques have been created and applied to the prediction of sgRNA activity in agronomy to discover sgRNAs with high on-target activity (Hesami et al., 2021; Niu et al., 2021). The creation of these techniques aids in determining the activity of intended sgRNA in crops and helps prevent unanticipated outcomes. Numerous studies have mentioned that they did not find any off-target activity in possible off-target locations. The off-target editing is insignificant and much less common when gRNAs are carefully generated than naturally occurring variability in plants.

In order to create rapeseed variants with less regulatory restrictions, it is also necessary to develop transgene-free genome editing techniques in this plant. A recent approach for efficiently delivering CRISPR complexes to rapeseed protoplasts using transient transfection has been discovered by researchers (Li et al., 2021). Other rapeseed researchers will benefit greatly from the advice provided by this improved protoplast regeneration methodology. Additionally, by infecting pollen with magnetic nanoparticles and then using that pollen to pollinate plants, transgenic cotton was effectively produced. Other nanoparticles, such as DNA origami and DNA nanostructures, as well as carbon nanotubes have also been successfully tested for the unaided transport of exogenous DNA (Zhao et al., 2017; Demirer et al., 2019; Kwak et al., 2019). If CRISPR/Cas9 for rapeseed genome editing could effectively use delivery by nanomaterials without tissue culture, it would be a quick way to create non-GM rapeseed while evading stringent GM laws. The CRISPR/Cas9 technology will produce additional top-notch rapeseed germplasm resources and provide enormous economic value with future advancement and the progressive resolution of remaining difficulties.

## Potential future prospects

In order to effectively utilize this technology for seed oil improvement in *Brassica napus*, we seek to present a concise description of the developments in CRISPR-mediated genome editing technology, as well as its applications and potential future prospects. The further advancements in CRISPR/Cas technology, coupled with other breakthroughs, will significantly benefit in the genetic improvement and molecular breeding of Brassica crops in terms of seed oil improvement and other agronomic traits. More gene targets for CRISPR/Cas9-based crop improvement will be discovered with the aid of current multiple omics, such as whole genome sequencing, RNA-seq, and small RNA-seq. The advent of straightforward, precise, and genome-editing tools using CRISPR/Cas9 has revolutionized crop breeding and plant biology research. In addition, new applications for CRISPR/Cas technologies will be created, with the potential to change the tempo and focus of brassica research. Improvements in *Brassica napus* basic genetic research, the development of innovative delivery methods, boosting public confidence in the security of CRISPR-modified crops, and the development of supporting regulatory frameworks are just a few of the many more successes that are needed in the future. In order to promote functional genomics and aid in the breeding of new Brassica varieties with improved seed oil characteristics, we believe that CRISPR/Cas technologies will be extensively used in this crop species.

## Conclusion

With an emphasis on the most recent discoveries in precise genome editing, we discuss current developments in the use of CRISPR/Cas technology in this mini-review. We also provide an outline of how *Brassica napus* traits for seed oil and fatty acid composition were altered using CRISPR/Cas technology. Finally, we discuss the challenges and future opportunities for the broad use of these technologies in *Brassica napus*. It will bolster the confidence of researchers studying rapeseed gene function and genetic improvement using genome editing technology to talk about the current state of application for novel, precise genome editing technology in plants as well as a number of potential limitations and technical hurdles.

## Author contributions

EA: presented the idea and design the project, and write the manuscript KZ: Revised it critically, Figure creation. All authors contributed to the article and approved the submitted version.
